# Novel airway smooth muscle–mast cell interactions and a role for the TRPV4-ATP axis in non-atopic asthma

**DOI:** 10.1183/13993003.01458-2019

**Published:** 2020-07-02

**Authors:** Sara J. Bonvini, Mark A. Birrell, Eric Dubuis, John J. Adcock, Michael A. Wortley, Pauline Flajolet, Peter Bradding, Maria G. Belvisi

**Affiliations:** 1 Respiratory Pharmacology Group, Airway Disease, National Heart and Lung Institute, Imperial College London, London, UK; 2 Research and Early Development, Respiratory and Immunology, BioPharmaceuticals R&D, AstraZeneca, Gothenburg, Sweden; 3 Dept of Infection, Immunity and Inflammation, University of Leicester University, Institute for Lung Health, Glenfield Hospital, Leicester, UK; 4 Contributed equally

## Abstract

Mast cell–airway smooth muscle (ASM) interactions play a major role in the immunoglobulin (Ig)E- dependent bronchoconstriction seen in asthma but less is known about IgE-independent mechanisms of mast cell activation. Transient receptor potential cation channel, subfamily V, member 4 (TRPV4) activation causes contraction of human ASM *via* the release of cysteinyl leukotrienes (cysLTs) but the mechanism is unknown. The objective of the present study was to investigate a role for IgE-independent, mast cell–ASM interaction in TRPV4-induced bronchospasm.

Bronchoconstriction was measured in anaesthetised guinea pigs and contraction of human and guinea-pig airway tissue assessed using isometric tension measurements. Increases in intracellular [Ca^2+^] were imaged using the Ca^2+^-sensitive dye FURA2, and time-lapse ptychography was utilised as a surrogate for contraction of ASM cells.

The TRPV4 agonist GSK1016790A caused contraction *in vivo* in the guinea pig, and in human and guinea-pig tracheal tissue, which was inhibited by the TRPV4 antagonist GSK2193874. GSK1016790A increased [Ca^2+^]_i_ and released ATP in human ASM cells without causing contraction. TRPV4 and ATP evoked contraction in isolated tracheal tissue but co-culture experiments indicated a requirement for human lung mast cells. Expression profiling and pharmacological studies demonstrated that mast cell activation was dependent upon ATP activating the P2X4 receptor. Trypsin was shown to evoke contraction of tracheal tissue *via* activation of PAR-2-TRPV4-ATP-cysLT axis indicating the potential disease relevance of this signalling pathway.

TRPV4 activation increases [Ca^2+^]_i_ and releases ATP from ASM cells triggering P2X4-dependent release of cysLTs from mast cells resulting in ASM contraction. This study delineates a novel mast cell–ASM interaction and TRPV4 as a driver of IgE-independent mast cell-dependent bronchospasm.

## Abstract 2

**A technique not previously applied to respiratory research now uncovers important IgE-independent mechanisms involved in human mast cell–airway smooth muscle interactions that may be responsible for the bronchospasm associated with non-atopic asthma** http://bit.ly/2U1n5nT

## Introduction

Asthma is a chronic airway inflammatory disease characterised by airflow obstruction and symptoms, such as chest tightness, wheezing and cough [1, 2]. Most asthmatics with early onset disease are atopic and demonstrate an allergic inflammatory response. This is referred to as T2 asthma and is driven by CD4+ T-helper (Th) 2 lymphocytes, mast cells and possibly innate lymphoid cells (ILC2) which secrete interleukin (IL)-4, IL-5 and IL-13 leading to eosinophilic inflammation and production of immunoglobulin (Ig)E from B cells [3, 4]. In atopic asthma, mast cells are sensitised by the binding of IgE to high-affinity IgE receptors (FCεR1). When allergen-specific IgE is cross linked by the relevant allergen, receptor aggregation triggers mast cell mediator release, with the release of preformed granule-derived mediators, prostanoids, cysteinyl leukotrienes (cysLT) and cytokines thought responsible for the early and late bronchospasm seen following allergen exposure [4]. Although atopic asthma and the T2 inflammatory response are often suppressed in patients treated with inhaled corticosteroids (ICS) subgroups of patients with both atopic and non-atopic asthma have persistent symptoms despite high doses of ICS [5] and so novel approaches to treatment are required.

Mast cells are likely key effectors in the pathogenesis of asthma through their association with the airway smooth muscle (ASM). In asthma, mast cells infiltrate the ASM bundles [6, 7], where they interact with ASM cells [8–11]. Human lung mast cells, *in situ* and when in co-culture with human ASM cells (HASMC), are in a continuously “activated” state with evidence of ongoing degranulation [7, 9, 11] and the expression of T2 cytokines [12] which can lead to increased inflammation and bronchoconstriction. However, it is recognised that there are multiple IgE-independent mechanisms of mast cell activation which may also play a role in asthma although less is known about the mechanisms involved. Here, we have studied the mast-cell–ASM interactions that are involved in IgE-independent bronchospasm elicited by transient receptor potential cation channel, subfamily V, member 4 (TRPV4) and by disease-relevant mediators, such as trypsin that can evoke contraction *via* proteinase-activated receptor-2 (PAR2) induced gating of TRPV4.

TRPV4 is a Ca^2+^-permeable, polymodally gated ion channel [13–15] which is expressed by and causes Ca^2+^ flux in HASMCs [16]. Recently, TRPV4 was shown to cause contraction of isolated guinea pig and human tracheal tissue *via* release of cysLTs [17]. In other cellular systems in the lung, TRPV4 activation evokes ATP release and initiates downstream functional consequences *via* activation of P2X receptors [18, 19]. ATP levels are increased in the bronchoalveolar lavage fluid (BALF) of asthmatics and in murine models of asthma [20]. Further, ATP induces mediator release in rodent mast cells independently of IgE [21, 22], and activation of purinoceptors present on rodent mast cells can lead to degranulation, cytokine secretion, chemotaxis and apoptosis [22]. Several P2X receptors are expressed on human lung mast cells (HLMCs) [23], and so we hypothesised that the TRPV4–ATP axis plays a role in human ASM–HLMC crosstalk, evoking mast cell-dependent bronchospasm which may play a key role in asthma pathophysiology.

## Methods

Further details of methods used can be found in the supplementary material.

### Animals

Male Dunkin–Hartley guinea pigs (300–800 g) were purchased from B&K (Hull, UK), and housed in temperature-controlled (21°C) rooms with food and water freely available for at least 1 week before commencing experimentation. Experiments were performed in accordance with the UK Home Office guidelines for animal welfare based on the Animals (Scientific Procedures) Act of 1986 and the ARRIVE guidelines [24].

### *In vivo* measurement of bronchoconstriction

Guinea pigs were anaesthetised with urethane (1.5 g·kg^−1^) and the trachea cannulated with a short length of Perspex tubing and animals artificially ventilated as previously described [18].

### Human tissue

Human airway samples (trachea, major bronchus, secondary bronchi) surplus to transplant requirement were obtained from the International Institute of Advancement of Medicine (IIAM, Edison, New Jersey, USA). We receive human tracheal and lung samples that are unsuitable for transplant, and therefore we often receive donor tissue that is not defined as “normal”. This is one of the limitations of this study but to avoid erroneous results we have used 13 donors and a range of techniques all of which confirm the hypothesis presented. We have been transparent about the provenance of the tissue by noting the lung diseases and smoking history of the donors in table 1. In all cases consent was granted for use in scientific research and ethical approval was obtained from the Royal Brompton & Harefield Trust (Ethics Number: REC 09/H0708/72, active from Dec 2009). See table 1 for the patient demographic details made available to us.

**TABLE 1:** Human tissue demographics for the donor lungs from which the tracheal and bronchi strips were sourced

**Patient**	**Sex**	**Age years**	**Respiratory conditions**
**1**	Female	31	Mild asthma
**2**	Male	48	Ex-smoker
**3**	Female	68	NA
**4**	Female	73	NA
**5**	Female	70	Smoker
**6**	Male	56	NA
**7**	Female	75	Asthma
**8**	Female	59	Bronchitis
**9**	Male	43	Smoker
**10**	Female	38	Asthma, bronchitis, smoker
**11**	Female	39	Asthma
**12**	Male	57	Smoker
**13**	Female	73	Ex-smoker

NA: not applicable.

### Cell culture

HASMCs were harvested as previously described [25]. Human lung mast cells (HLMCs) from the University of Leicester were purified from macroscopically normal lung resections from three patients as described previously [26] with a final purity greater than 99%. The purified HLMCs were cultured in DMEM/HEPES containing Glutamax I and 10% heat inactivated fetal calf serum (FCS), 100 ng·mL^−1^ stem cell factor, 50 ng·mL^−1^ IL-6 and 10 ng·mL^−1^ IL-10 with half the medium replaced every 7 days [27].

### *In vitro* measurement of contraction: organ bath

Human tracheal and bronchial tissue (approximately 3–4 mm in width) and guinea-pig trachea (approximately two cartilage rings in width) was cut longitudinally by cutting through the cartilage directly opposite to the smooth muscle layer and transverse sections cut to produce strips and connected to force transducers as previously described [28].

### Calcium imaging in HASMCs

HASMCs harvested from donor tissue were grown in 35 mm glass bottom fluorodishes in DMEM supplemented with 10% FCS, and serum starved 24 h prior to experimental use. On the day of the experiment, DMEM was removed from the cells and replaced with sterile extracellular solution (ECS) and allowed to equilibrate for 30 min at 37°C. The cells were then loaded with the intracellular calcium dye Fura-2-AM (12 µM, supplemented with 1% PowerLoad+Probenicid (1 mM)) for 1 h and [Ca^2+^]_i_ responses monitored using a Widefield inverted microscope.

### Assessment of single HASM cell contraction: ptychography

A novel imaging technique, ptychography, was utilised as a surrogate for cellular contraction of HASMCs [29]. Ptychography is a label-free, high-contrast microscopy technique which yields contrast similar to fluorescent imaging with minimal cell manipulation. The dish containing the cells and a low intensity near infrared laser (635 nm) are moved over each other to create a sequential array of overlapping illuminated areas, where the light scattered is captured as an array of diffraction pattern on a detector. This diffraction pattern array is then processed using the Virtual Lens algorithm to calculate a quantitative measure of the light absorbed, the scattering and the phase delay introduced into the illumination as it passes through the specimen, which enables analysis of the three-dimensional characteristics of the sample [29]. This technique has yielded contractile responses in HASMCs (for example, in response to acetylcholine (ACh)) matching [Ca^2+^]_i_ elevation recorded in the same cells and similar to contractile responses found using whole tissue [30].

### Data analysis

Data were expressed as mean±sem of n observations. Statistical significance was determined using students t-test or one-way ANOVA with an appropriate *post hoc* test. Statistical significance was set at p<0.05 and all treatments were compared with the appropriate vehicle control and n numbers refer to the number of different donor tissues/cells used.

## Results

### Effect of TRPV4 on contraction *in vivo*

**FIGURE 1:**
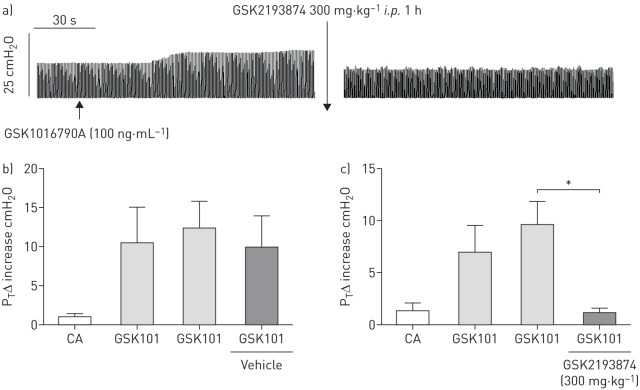
a) Example trace of a GSK1016790A 100 ng·mL^−1^ (153 nM for 15 s) mediated response. Contraction was unchanged following vehicle (6% cyclodextrin in saline) administration (b) but significantly inhibited following administration of GSK2193874 (300 mg·kg^−1^, i.p.) (c). Data are presented as mean±sem of n=3. *: p<0.05, paired *t*-test comparing responses before and after antagonist/vehicle administration in the same animal.

Aerosolised GSK1016790A 100 ng·mL^−1^ (153 nM for 15 s) caused a significant and sustained increase in tracheal pressure in anaesthetised guinea pigs (figure 1a–c). In all cases, both vagal nerves were cut, therefore this effect was due to a direct effect on the smooth muscle rather than a parasympathetic reflex. Increases in tracheal pressure induced by a submaximal concentration 100 ng·mL^−1^ (153 nM for 15 s) of the TRPV4 agonist GSK1016790A were significantly inhibited 1 h following *intraperitoneal* administration of the TRPV4 antagonist GSK2193874 (300 mg·/kg^−1^; 6% cyclodextrin in saline) (figure 1c) but remained unaffected following vehicle (figure 1b).

### Effect of TRPV4 agonists on isolated airway tissue *in vitro*

**FIGURE 2:**
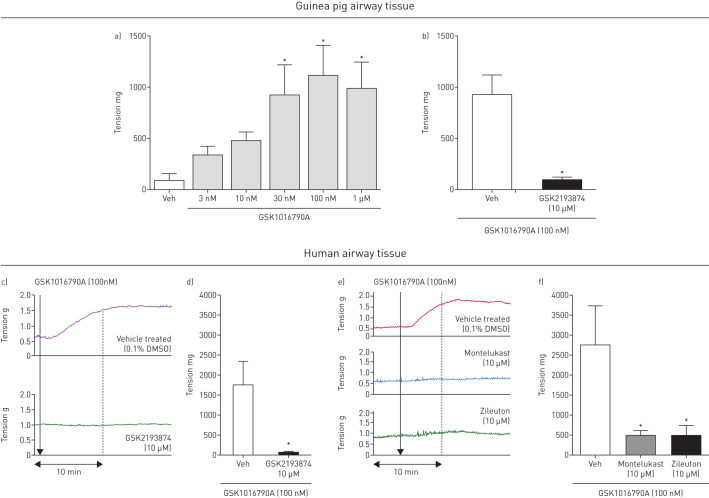
GSK1016790A caused contraction in isolated guinea pig trachea (a), which was inhibited by GSK2193874 (10 µM) (n=4–6) (b). c) Response to GSK1016790A (100 nM) plus vehicle (top panel) or GSK2193874 (10 µM) (bottom panel) in human tissue. d) GSK2193874 (10 µM) inhibited the response in human tissue (n=3). (f) Preincubation with either montelukast (10 µM) (example trace (e), middle panel), or zileuton (10 µM) (example trace (e), bottom panel) significantly inhibited GSK1016790A induced contraction of human tissue (n=3). Data are presented as mean±sem *: p<0.05 using a one-way ANOVA with Dunnett's multiple comparison test comparing responses to vehicle (a, f) or a t-test comparing responses with antagonist to the vehicle control (b, d).

GSK1016790A caused a concentration-dependent contraction in isolated guinea pig trachea. Due to the long-lasting contraction induced by the agonist, only one concentration was tested on each piece of tissue (figure 2a). Contraction was significantly inhibited following 30 min preincubation with GSK2193874 (10 µM) (figure 2b). A similar effect was seen in human tissue (figure 2c and d). A recent publication indicated that TRPV4-induced contraction of ASM is dependent upon the release of cysLTs [17]. Consistent with this observation, GSK1016790A (100 nM)-induced contraction was inhibited following preincubation with a cysLT1 antagonist (montelukast 10 µM) and a 5-LO inhibitor (zileuton 10 µM) in human tissue, confirming that cysLTs are responsible for the contraction induced by the TRPV4 agonist in human ASM (figure 2e and f). Monteleukast (10 µM) and zileuton (10 µM) alone had no effect on airway tone *per se*.

### The role of mast cells in TRPV4 induced contraction

**FIGURE 3:**
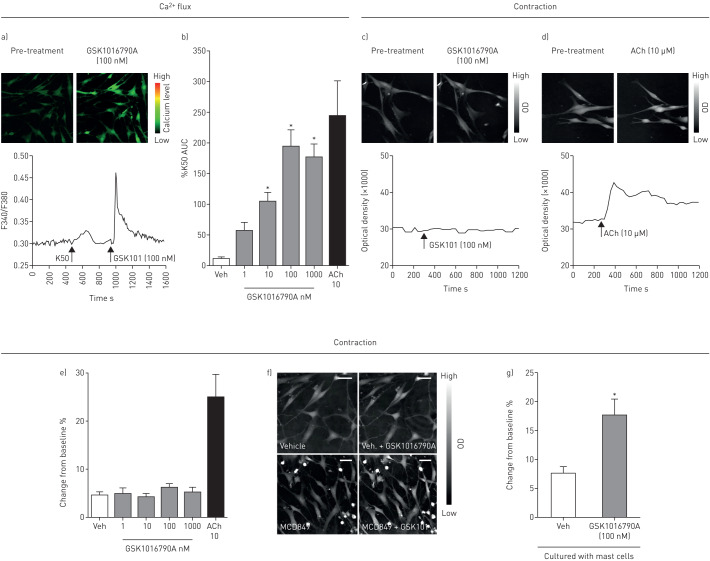
a and b) Example image, trace and graph of the GSK1016790A-induced increase in calcium in human airway smooth muscle cells (HASMCs). Example ptychography reconstituted images (top panel) and traces (bottom panel) from (c) GSK1016790A (100 nM) treated and (d) acetylcholine (Ach) (10 µM) treated HASMCs (n=2 donors). e) Ptychography data indicating no contractile effect to GSK1016790a in human HASMCs alone. ACh was used as a positive control (n=2 donors). f) Reconstituted ptychographic images; top two panels show HASMCs alone. Bottom two panels show HASM co-cultured with mast cells, where the addition of GSK1016790A (100 nM) led to an increase in optical density. g) In the presence of mast cells, GSK1016790A (100 nM) caused contraction of HASMCs (n=2 donors for HASMCs, n=3 donors for human lung mast cells (HLMCs)). Data are presented as mean±sem (n=8–56 readings;). Scale bar indicates 100 µm. *: p<0.05 using a Kruskall Wallis test with Dunn's post comparison test comparing responses to vehicle (b) or a *t*-test comparing responses to vehicle control (g).

The TRPV4 agonist GSK1016790A caused a concentration-dependent increase in [Ca^2+^]_i_ in HASMCs as assessed by fluorescent FURA2 imaging (figure 3a and b). To assess the effect of GSK1016790A on contraction of primary HASMCs, we used a novel imaging technique known as ptychography. Using this technique, GSK1016790A incubated with HASMCs alone did not cause contraction; however, the positive control ACh (10 µM) did, as shown by an increase in optical density (figure 3c, d and e). Mast cells are a major source of cysLTs and in co-cultures of HASMCs with primary HLMCs, GSK1016790A caused contraction of the HASMCs as demonstrated using ptychography. However, mast cell-free media had no effect, indicating the requirement for HLMCs for TRPV4 mediated contraction of HASMCs (figure 3e, f and g; a video showing a HASM cell contracting in the presence of HLMCs and GSK1016790A can be found in the supplementary material).

### Role of ATP in TRPV4 induced contraction

**FIGURE 4:**
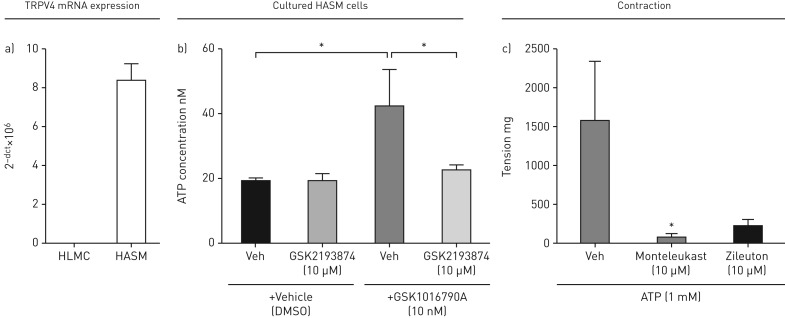
a) TRPV4 mRNA is expressed on human airway smooth muscle cells (HASMCs), but not on human lung mast cells (HLMCs). b) GSK1016790A (100 nM) induced ATP release from HASMCs which was inhibited by GSK2193874 (10 µM). c) ATP contraction of isolated human tracheal strips was inhibited by montelukast and zileuton (10 µM). Data are shown as mean±sem. HASMCs were isolated from 4 patients, tracheal strips from 3 donors' lungs. *: statistical significance (p<0.05); t-test comparing responses to relevant control (b) or using a one-way ANOVA with Dunnett's multiple comparison test comparing responses to vehicle (c).

The data suggested that TRPV4 agonists activate the TRPV4 ion channel present on HASMCs to increase [Ca^2+^]_i_ causing the release of an unknown mediator that would cause the release of cysLTs from mast cells. HASM cells, but not HLMCs, expressed TRPV4 at the mRNA level (figure 4a), suggesting that activation of TRPV4 does not have a direct effect on mast cells. Previous work has shown that activation of TRPV4 on macrophages and airway sensory nerves induces the release of ATP [18, 19], suggesting that ATP may be the previously unknown mediator. GSK1016790A caused ATP release from cultured human ASM cells, which was inhibited following administration of GSK2193874 (10 µM) (figure 4b). ATP (1 mM) was shown to cause contraction of human isolated smooth muscle in the organ bath, which was inhibited by both montelukast and zileuton, indicating a similar pharmacological profile to GSK1016790A (figure 4c). Furthermore, ATP caused the release of cysLTs from human donor lung mast cells but did not contract HASM cells that were cultured alone and in the absence of HLMCs (supplementary figure E1a and b).

### Mechanism of TRPV4 induced contraction of human ASM

**FIGURE 5:**
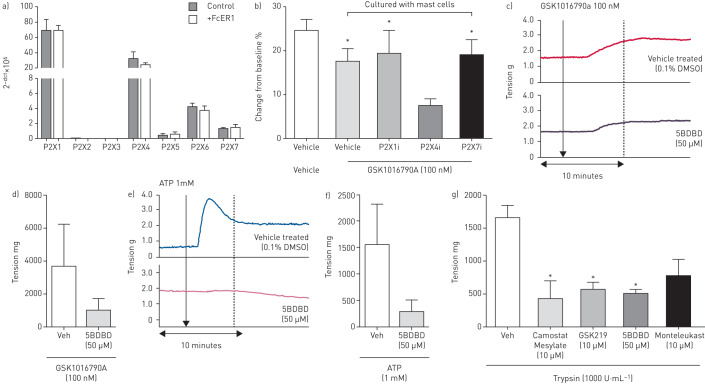
a) Expression of P2X receptor mRNA on human lung mast cells (HLMCs) (n=3 donors). b) Effect of P2X receptor antagonists P2X1 (Ip51, 10 µM), P2X4 (5BDBD, 50 µM) and P2X7 (AZ11645373, 10 µM) on contraction of human airway smooth muscle cells (HASMCs) treated with GSK101679A (100 nM). Effect of the P2X4 inhibitor (5BDBD, 50 µM) on GSK101679A (100 nM) (c and d) and ATP (1 mM) (e and f) -induced contraction of human tissue (n=3). g) Trypsin induced contraction of human tracheal strips inhibited by camostat mesylate, GSK2193874, montelukast and 5BDBD (n=2–3). Data are presented as mean±sem. *: p<0.05 t-test compared with relevant control (b) or using a one-way ANOVA with Dunnett's post test (p<0.05) comparing antagonist with vehicle control (g).

ATP activates both ionotropic P2X purinoceptors and metabotropic P2Y receptors. Mast cells have been shown to express a number of purinoceptor ion channels [23, 31] and P2X1, P2X4, P2X5, P2X6 and P2X7 were shown to be expressed at the mRNA level on primary HLMCs (figure 5a). Using ptychography, contraction of the human ASM cells induced by GSK1016790A (100 nM) in the presence of HLMCs was only significantly inhibited following incubation with the P2X4 antagonist 5BDBD (50 µM) but not following incubation with a P2X1 (Ip5I 10 µM) or a P2X7 antagonist (AZ11645373 10 µM) (figure 5b). This result translated in human airway tissue, where contraction induced by both GSK1016790A (100 nM; figure 5c and d) and ATP (1 mM; figure 5e and f) was inhibited following incubation with 5BDBD (50 µM).

In the search for an endogenous TRPV4 ligand, we speculated that PAR2 activation may play a role. The G protein-coupled receptor (GPCR) PAR2 has been shown to be functionally coupled to TRPV4 and cause activation of the ion channel [32, 33]. Using rtPCR, similarly to TRPV4, PAR2 was shown to be consistently expressed on HASMs, but not HLMCs, where it was only expressed in one-third of patients (supplementary figure E2a). We then utilised the endogenous PAR2 ligand trypsin (1000 U·mL^−1^), which caused contraction of isolated human tracheal strips (figure 5g) as previously described [34]. This contraction was inhibited by the same antagonists that blocked TRPV4-induced contraction, including GSK2193874, montelukast and 5BDBD, along with the serine protease inhibitor camostat mesylate (figure 5g). These data indicate that asthma “disease-relevant” mediators such as mast-cell proteases (*e.g.* PAR2 activators such as tryptase) can activate this pathway. This mechanism was shown to be TRPV4 contraction-specific as neither GSK2193874 (10 µM), montelukast (10 µM) nor 5BDBD (50 µM) had any effect on contraction induced by the common contractile stimuli histamine and ACh (supplementary figure E2b and c).

**FIGURE 6:**
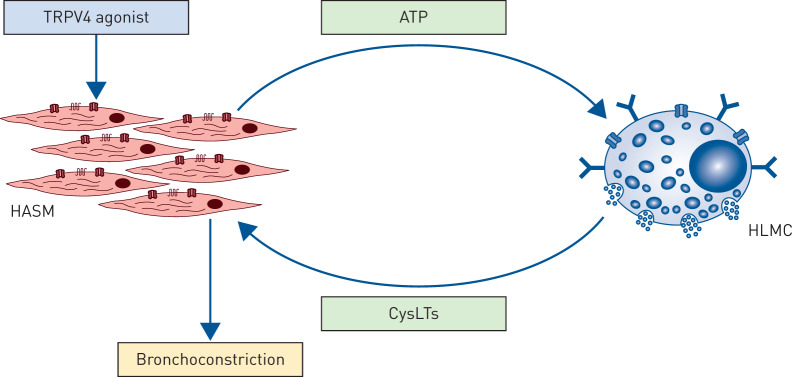
Schematic diagram which illustrates the mast cell dependent contraction of human airway smooth muscle cell (HASM) evoked by TRPV4. In this series of experiments, we have demonstrated that activation of TRPV4 on HASM induces the release of ATP which activates P2X4 receptors on mast cells, near and within the ASM bundles, to induce the release of cysteinyl leukotrienes which causes cystLT1-dependent contraction of ASM.

In this series of experiments, we have demonstrated that activation of TRPV4 on human ASM induces the release of ATP which activates P2X4 receptors on HLMCs in close proximity to the ASM, in turn inducing the release of cysLTs which causes cysLT1-dependent contraction of ASM. This mechanism is outlined as a schematic in figure 6.

## Discussion

HLMC–ASM interactions are thought to play a fundamental role in the pathogenesis of atopic asthma due to the IgE-dependent release of bronchoconstrictor mediators and cytokines which contribute to the early- and late-phase responses following allergen exposure. However, it is now recognised that there are many IgE-independent mechanisms of mast cell activation which may play a role in both atopic and non-atopic asthma and across other chronic lung diseases [4]. Here we investigate the role that ASM–mast cell interactions and non-IgE-dependent mechanisms play in contractile responses to “disease-relevant” asthma mediators.

Mast cell proteases are believed to play an important role in the development of asthma and the continuing symptoms that are seen with persistent disease [4]. Many of the proteases associated with allergens or released from mast cells are serine proteases (trypsin-like, chymotrypsin-like or neutrophil elastase-like) that can activate the GPCR, PAR2. The effects of PAR2 in both allergic and also nonallergic asthma have been reported to be complex, with Nichols *et al.* [34] demonstrating that PAR2 can act *via* several independent signalling pathways; proinflammatory effects are thought to be mediated through β-arrestin and protective effects are mediated through GPCR activation and increases in intracellular calcium. However, it is not clear how PAR2 activation leads to functional consequences such as bronchospasm [35]. Signals that originate from the GPCR superfamily activation, including PAR2, have been shown to converge on certain transient receptor potential family members, including TRPV4, leading to channel activation and sensitisation which amplify pain, itch and neurogenic inflammation [32, 36]. TRPV4 activation contributes to a variety of effects within the lung, including sensory nerve activation, intracellular Ca^2+^ flux in ASM [16–18] and cysLT_1_-dependent contraction of both guinea pig and human airway tissue [17].

Here, we show for the first time, the ability of the TRPV4 agonist GSK1016790A to cause slow onset, sustained ASM contraction *in vivo* in the guinea pig which was abolished in the presence of a TRPV4 antagonist. *In vitro* this contraction was also slow in onset and long lasting and was inhibited by the TRPV4 inhibitor GSK2193874. As previously shown, we confirmed that this contraction was inhibited by the cysLT_1_ receptor antagonist montelukast, and the 5-LO inhibitor, zileuton, in both guinea pig and human tissue [17]. Since cysLTs are known to be synthesised by activated mast cells [17, 37], it was hypothesised that mast cell-ASM interactions were central to this process.

To investigate this interaction further, we initially investigated whether TRPV4 activation could evoke increases in [Ca^2+^]_i_ in isolated HASMCs as this assessment is often used as a surrogate measure of contraction [38]. The TRPV4 agonist GSK1016790A induced an increase in [Ca^2+^]_i_ in HASMCs as previously shown [16, 17] but did not cause contraction of ASM cells in culture as assessed by a novel technique; ptychography [30], which utilises changes in optical density as a surrogate for changes in contractile status. An increase in calcium is often suggestive of contraction of ASM cells; however, other stimuli have been shown to have a strong increase in intracellular calcium in ASM but do not initiate contraction, including bitter taste receptor (TASR) agonists [39], which instead induce relaxation. Mast cells are known to be resident within ASM bundles in both healthy, non-atopic patients [40] and those with asthma where they play a key role in contraction through the release of various mediators including cysLTs [6]. Therefore, HASMCs were co-cultured with primary HLMCs to assess the mast cell-dependent nature of the TRPV4-induced contraction. Using ptychography, we demonstrated that GSK1016790A was only able to increase optical density indicative of contraction when HASMCs were in the presence of HLMCs. This was unlikely a direct effect on HLMCs, as RT-PCR indicated that TRPV4 mRNA was expressed on HASMCs but not on HLMCs.

Since TRPV4 was expressed on HASMCs, but not on HLMCs, and because of the delayed contractile response we hypothesised the involvement of a secondary messenger. TRPV4 induces ATP release in several different cellular systems which can act on a number of purinoceptors that have a variety of downstream effects in the lung [18, 19]. Similarly, we found that the addition of the TRPV4 agonist to isolated HASMCs induced ATP release. ATP is a ubiquitous molecule found in every cell of the body, and directly activates animal mast cells [20–22]. We demonstrated that, consistent with previous studies, a number of purinoceptors were expressed on the cell surface of HLMCs [23]. In addition, we have demonstrated that contraction of airway tissue in response to ATP can be abolished in the presence of a P2X4 inhibitor, indicating that it is likely to be the P2X4 receptor present on HLMCs which is activated by ATP, and induces the release of cysLTs. P2X4 is the most widely expressed of the purinergic P2X receptors [41] and previous work has linked P2X4 with asthma, as the P2X4 inhibitor 5BDBD attenuated the response to antigen in an ovalbumin “asthma” model in BALBc mice [42, 43]. P2X4 is a Ca^2+^ permeable ion channel, and Ca^2+^ signalling plays a major role in regulating secretion from HLMCs [44]. This mechanism has also been linked with P2X7 activation which can cause degranulation of the LAD2 human mast cell line [31]. Elevated levels of ATP and its breakdown products have been found in the BAL fluid of asthmatic patients [20] and ATP can cause bronchoconstriction and dyspnoea [45], indicating that this may be a relevant pathway for mast cell activation and cysLT release in the asthmatic lung.

Identification of the PAR2-TRPV4-ATP axis in evoking bronchospasm illustrates the complex role that mast cells may have in driving the pathophysiology of structural cells and symptoms in both atopic and non-atopic asthma. These and similar mechanisms may be important in the generation of asthma symptoms in response to various stimuli including for example exercise induced bronchospasm (EIB). CysLTs are thought to be key mediators of EIB; they are released from activated mast cells, and asthmatics which exhibit EIB have been shown to have increased levels of cystLTs in both sputum and exhaled breath condensate [46]. Furthermore, a single oral dose of montelukast, which we have shown to inhibit TRPV4 mediated contraction, has been shown to exert significant protection against EIB at 2, 12 and 24 h post exercise [47]. The symptoms of EIB are thought to be evoked by the osmotic and thermal effects of increased respiration [46] which are known to activate TRPV4 [48]. In addition, tryptase has also been shown to be elevated in asthmatic patients with EIB [49] providing further evidence of a role for this axis in exercise-induced bronchospasm.

Mast cells appear to be key effector cells in the pathogenesis of asthma through their close association with the ASM. However, the functional consequences of this close association are not fully understood. In this study we have implicated the PAR2-TRPV4-ATP-cysLT axis in mast cell-dependent bronchospasm. TRPV4 activation is linked to the GPCR, PAR2, where activation of PAR2 has been shown to both sensitise [36] and also directly gate TRPV4 [32, 50]. In support of this hypothesis we have shown that trypsin can cause contraction of human tracheal strips *via* activation of PAR2-TRPV4-ATP-cysLT axis, as the contraction was inhibited by the serine protease inhibitor camostat mesylate [51], the TRPV4 antagonist GSK2193874, the P2X4 inhibitor 5BDBD and also montelukast.

In conclusion, we have described a novel mast cell-ASM interaction involving the ion channel TRPV4. We suggest that TRPV4 is activated on ASM by endogenous ligands such as arachidonic acid derivatives or proteases, leading to the release of ATP, potentially through the pannexin ion pore, which activates P2X4. This mechanism may play an important role in IgE-independent mast cell mediator release in asthma, for example during EIB, and in non-atopic asthma where common aeroallergens do not appear to contribute but levels of endogenous indirect activators of TRPV4 such as tryptase are elevated [49] and also where cysLT_1_ receptor antagonists such as montelukast have been shown to be effective [52]. Furthermore, this mechanism may also be relevant in other lung diseases where increased lung tissue mast cells have been identified and linked with disease pathophysiology [53].

## Supplementary material

10.1183/13993003.01458-2019.Supp1 **Please note:** supplementary material is not edited by the Editorial Office, and is uploaded as it has been supplied by the author.
Supplementary material. ERJ-01458-2019.SUPPLEMENT
Supplementary figure E1. a) The effect of ATP on contraction of primary HASM cells alone. ATP at 0.3, 1 or 3 mM did not cause contraction of the cells analysed using ptychography, in some cases there was relaxation. The contractile stimulus ACh (10 μM) caused contraction of the cells as expected. Data shown as % change from baseline (resting state), N=2 patients, n=17–27 cells. b) The effect of vehicle, ATP at 1 mM and 3 mM and PMA (10 nM) ionomycin (1 μM) on total cysteinyl leukotriene release from primary human mast cells. Addition of ATP (1 or 3 nM) caused a concentration dependent increase in total cysteinyl leukotriene release compared to vehicle control. Data shown is from mast cells isolated from 1 (73-year-old female donor). ERJ-01458-2019.FIGURE_E1
Supplementary figure E2. a) PAR2 mRNA was expressed in HASMCs from six patients, but not in HLMCs of two patients, and expressed at very low levels in the third. The effect of vehicle GSK2193874, montelukast and 5BDBD on contraction induced by the common contractile stimuli histamine (b) and acetylcholine (c) in isolated guinea pig trachea. No antagonist had any effect on contraction different to that of vehicle. Mean±SEM of n=6 (HASMs), 3 (HLMCs), 3–4 (trachea). ERJ-01458-2019.FIGURE_E2
Supplementary video. ERJ-01458-2019.VIDEO

## Shareable PDF

10.1183/13993003.01458-2019.Shareable1 This one-page PDF can be shared freely online.
Shareable PDF ERJ-01458-2019.Shareable

## Supplementary Material

ERJ-01458-2019.Shareable.pdf
