# Supplementary material for: Novel airway smooth muscle–mast cell interactions and a role for the TRPV4-ATP axis in non-atopic asthma
Source: Eur Respir J. 2020 Jul 2;56(1):1901458. doi: 10.1183/13993003.01458-2019 (PMC7330131; doi:10.1183/13993003.01458-2019)
Supplement: Supplementary file 1 [file ERJ-01458-2019.SUPPLEMENT.pdf]

ONLINE REPOSITORY: Novel airway smooth muscle-mast cell interactions and a role for the TRPV4-ATP axis

*Sara J. Bonvini PhD[1], *Mark A. Birrell PhD[1,2], Eric Dubuis PhD[1], John J. Adcock PhD[1], Pauline Flajolet PharmD[1], Michael A. Wortley PhD[1], Peter Bradding[3], #Maria G. Belvisi PhD[1,2].

Affiliations:

[1]Respiratory Pharmacology Group, Airway Disease, National Heart & Lung Institute, Imperial College London, Exhibition Road, London SW7 2AZ, UK; [2] Research & Early Development, Respiratory, Inflammation Autoimmunity (RIA) BioPharmaceuticals R&D , AstraZeneca, Gothenburg, Sweden, [3]Department of Infection, Immunity and Inflammation, University of Leicester University, Institute for Lung Health, Glenfield Hospital, Leicester, LE3 9QP, UK.

* Contributed equally

# Correspondence to: Professor Maria G. Belvisi

Author Contributions: Conception and design: MAB, MGB, SJB; data analysis and interpretation: MGB, MAB, SJB, ED, JJA, MAW, PF, PB; writing the paper: SJB, MAB, MGB.

Funding: SJB and ED was funded by a Medical Research Council (MRC, UK) MICA award (MR/K020293/1) and Wellcome Trust (207504/B/17/Z). MAW was funded by the North-West Lung Centre Charity. The human vagus experiments in this study were undertaken with the support of the NIHR Respiratory Disease Biomedical Research Unit at the Royal Brompton and Harefield NHS Foundation Trust. Work in Leicester was supported by the National Institute for Health Research Leicester Biomedical Research Centre - Respiratory. The views expressed are those of the author(s) and not necessarily those of the NHS, the NIHR or the Department of Health.

METHODS

In vivo measurement of bronchoconstriction

Guinea pigs were anaesthetized with urethane (1.5g/kg) and the trachea cannulated with a short length of Perspex tubing and animals artificially ventilated as previously described[1]. The right jugular vein and carotid artery (passed to the ascending aorta/aortic arch) were also cannulated for drug administration and measurement of systemic arterial blood pressure. Animals were paralysed with vecuronium bromide (0.1mg/kg i.v. initially then 0.05mg/kg i.v. every 20 minutes to maintain paralysis). The cervical vagal nerves were located and both cut at the central end[19]. Tracheal pressure was measured with an air pressure transducer (SenSym647) connected to a side arm of the tracheal cannula.

GSK1016790A (100ng/ml, 153nM for 15s) was aerosolised into the airways and changes in bronchoconstriction indicated by an increase in tracheal pressure which were monitored for 60 minutes or until responses returned to baseline levels. In antagonist studies, GSK2193874 (300mg/kg, i.p.) or vehicle (6% cyclodextrin in saline, i.p) were administered 30 minutes prior to aerosolization of GSK1016790A (100ng/ml).

Cell culture

Primary HASMCs were harvested as previously described[2]. Once extracted, cells were cultured in Dulbecco Modified Eagles medium (DMEM) containing L-glutamine (2mM), sodium pyruvate (1mM), penicillin (100U/mL), streptomycin (100µg/mL), amphotericin B (5µg/ml), nonessential amino acids (1%) and supplemented with 10% (v/v) foetal bovine serum (FBS). The HASMCs were cultured in 35mm glass bottom fluorodishes in air containing 5% carbon dioxide in a humidified chamber at 37°C, until cells had grown to 50% confluence. Prior to treatment, culture media was aspirated, and replaced with serum-starved media (DMEM containing L-glutamine (2mM), sodium pyruvate (1mM), penicillin

(100U/mL), streptomycin (100µg/mL), amphotericin B (5µg/ml), nonessential amino acids (1%), and Bovine Serum Albumin (1.3%) for 24h to induce growth to arrest.

Expression profiles in HASMCs and HLMCs

As a number of TRPV channels have previously been shown to be expressed in both guinea pig and human ASM, including TRPV1 and TRPV2 alongside TRPV4[3,4], expression of TRPV4 on ASM was confirmed using Quantitative Real time PCR. Assays for the TRP and P2X ion channels and the PAR2 GPCR were purchased from Applied Biosystems and validated using cDNA from human tissue/organs that expressed the target ion channel at high levels. Expression levels of TRP and P2X ion channels along with PAR2 were determined using the validated assays, with real time RT-PCR performed as described previously[5].

***In vitro* measurement of contraction: Organ Bath**

Human tracheal and bronchial tissue (approximately 3-4mm in width) and guinea-pig trachea (approximately 2 cartilage rings in width) was cut longitudinally by cutting through the cartilage directly opposite to the smooth muscle layer and transverse sections cut to produce tracheal strips. These were sutured and attached to a steel hook at the bottom end and to force-displacement transducers on the other end in 10ml organ baths containing Krebs-Hensleitt solution kept at 37°C, bubbled with 95% O2/5% CO_2. Changes in force were measured isometrically using force-displacement transducers connected to a data acquisition system as previously described[5]. In all cases the epithelium was left intact, and indomethacin (10 µM), a non- selective COX inhibitor was present throughout to prevent the production of endogenous prostanoids which are known to influence airway smooth muscle tone. Once the tissues had equilibrated they were challenged with a supramaximal concentration of ACh (1 mM) three times to confirm viability and to standardise responses. All experiments were carried out under basal levels of tension. Following the final ACh response, single concentrations of the TRPV4 agonist GSK1016790A (1 nM-1 µM), or ATP (100 µM-3

mM) were added to separate baths and left to incubate for 40 minutes, or until responses returned to baseline, after which a final stimulation with ACh was added to the baths to ensure tissue viability. For antagonist experiments, the tissue was preincubated with the selective antagonists (TRPV4: GSK2193874 (10 µM); CysLt1: Montelukast (10 µM); 5LO: Zileuton (10 µM); P2X4: 5BDBD (50 µM)) in separate baths for 30 minutes. A submaximal concentration of GSK1016790A (100 nM) or ATP (1 mM) was then added to the baths and left to incubate for 40 minutes, or until responses returned to baseline. A final stimulation with ACh was added following this to ensure tissue viability.

Calcium imaging in HASMCs

HASMCs harvested from donor tissue were grown in 35mm glass bottom fluorodishes in DMEM supplemented with 10% FCS. Twenty-four hours prior to experimental use, culture media was replaced with serum-starved media. On the day of the experiment, DMEM was removed from the cells and replaced with sterile extracellular solution (ECS) and allowed to equilibrate for 30 minutes at 37°C. The cells were then washed twice with ECS at 25°C and loaded with the intracellular calcium $[Ca^{2+}]_i$ dye Fura-2-AM (12µM, supplemented with 1% PowerLoad + Probenicid (1mM)) for 1h in the dark at RT. The cells were then washed twice with ECS and left to de-esterify in the dark at room temperature for 1h prior to imaging analysis. After washing, a single fluorodish was placed in a full incubation chamber mounted on a stage of a Widefield inverted microscope and imaged at excitation and emission fluorescence wavelengths of approximately λ=360/380 nm and λ=650 nm, respectively to measure $[Ca^{2+}]_i$ in response to agonist. K50 (50mM potassium solution, as opposed to 5.4mM used in ECS) was applied at the start of each experiment for 30s to assess cell viability and normalise responses. Once responses were obtained the cells were washed to return to baseline, and then treated with vehicle (0.1% DMSO) for ten minutes, then washed, following which a single concentration of the TRPV4 agonist (1-1000nM) was added to the dish and incubated for ten minutes and $[Ca^{2+}]_i$ responses monitored.

Assessment of single human airway smooth muscle cell contraction: Ptychography

A novel imaging technique, ptychography, was utilised as a surrogate for cellular contraction of HASMCs[7]. Ptychography is a label free, high contrast microscopy technique which yields contrast similar to fluorescent imaging with minimal cell manipulation[7]. The dish containing the cells and a low intensity near infrared laser (635nm) are moved over each other to create a sequential array of overlapping illuminated areas, where the light scattered is captured as an array of diffraction pattern on a detector. This diffraction pattern array is then processed using Virtual Lens© algorithm to calculate a quantitative measure of the light absorbed, the scattering and the phase delay introduced into the illumination as it passes through the specimen, which enables analysis of the 3D characteristics of the sample[7]. The parameter extracted for this study was the optical density which measures the thickness of the cells as it changes during isotonic contraction. This technique has yielded contractile responses in HASMCs (for example in response to ACh) matching $[Ca^{2+}]_i$ elevation recorded in the same cells and similar to contractile responses found using whole tissue[8].

In order to determine the role of mast cells in TRPV4 induced contraction, HASMCs were cultured with HLMCs, or mast cell media (DMEM/HEPES containing Glutamax I and 10% heat inactivated fetal calf serum, 100ng/ml SCF, 50ng/ml interleukin-6 and 10ng/ml interleukin-10). Cultured primary HASMCs were growth arrested for 24 h, following which they were co-cultured with HLMCs +3% FBS at a 4:1 ASM to mast cell ratio, or mast cell media, in triplicate in 35mm glass bottomed fluorodishes. One hour later, cells were treated with either vehicle or P2X antagonist and incubated at 37°C for 1 hour. Cells were placed in the ptychography system (VL21, Phase Focus UK) and then stimulated with GSK1016790A (100nM) or vehicle (0.1% DMSO). The effect of GSK1016790A on contraction of HASMCs alone and HASMCs co-cultured with HLMCs was then assessed using time lapse ptychography. A control experiment conducted in parallel was HASMCs cultured in the same conditions without HLMCs, and treated with either GSK1016790A or ATP.

ATP release assay

Human ASM cells from four patients were seeded onto separate 24 well plates and grown to 90% confluence. 24 hours following growth arrest, cells were incubated with vehicle or GSK2193874 (10µM) at 37°C for 30 minutes. The cells were then incubated with GSK1016790A (10nM) or vehicle for 1 hour at 37°C, following which the supernatant was aspirated and frozen for later analysis. ATP levels were assessed using an ATPlite assay (Perkin Elmer, Cambridge, UK) according to pack instructions.

Cysteinyl Leukotriene ELISA

Primary mast cells were isolated from a macroscopically normal lung biopsy (n=1) as described previously[9]. Cells were cultured for 4 weeks in DMEM GlutaMAX containing 10%FCS, hSCF (100ng/mL), hIL-6 (50ng/mL) and hIL-10 (10ng/mL) prior to any experiment. Final purity was >98% assessed by metachromatic staining with modified Wright-Giemsa stain.

Cells were seeded onto a 96 well plate at a density of 10,000 cells/well in 100µL DMEM GlutaMAX containing 10%FCS, SCF (100ng/mL), IL-6 (50ng/mL) and IL-10 (10ng/mL). 24 hours later, cells were washed twice and equilibrated for 10min at 37°C in 90uL HEPES containing 0.04% BSA SCF (100ng/mL), IL-6 (50ng/mL) and IL-10 (10ng/mL) prior to challenge with vehicle (1% dH_20), ATP (1mM), ATP (3mM), or the positive control positive control, PMA (10nM)/ionomycin (1µM) in duplicate. Following a 30-minute incubation, the supernatant was aspirated and frozen for later analysis. Total Cysteinyl Leukotriene (LTC_4, LTD_4 and LTE_4) levels were assessed using a Cysteinyl Leukotriene Express ELISA kit (Cayman Chemical) according to pack instructions. Viability of the cells was not affected, with >90% in all treatment groups.

Compounds and materials

The selective TRPV4 agonist and antagonist GSK1016790A and GSK2193874 were chosen as the ligands to probe the role of the TRPV4 receptor as they have been shown to be potent activators and

inhibitors both *in vivo* and *in vitro*, with limited off target effects[10,11]. These were purchased from Sigma Aldrich (Poole, UK) with montelukast, zileuton, AZ11645373 and DMSO. 5-BDBD, and Ip5I were purchased from Tocris (UK). Drugs were made up in stock solutions using DMSO, or dH_20 (ATP) with the final concentration of DMSO or dH_20 kept at 0.1% for experiments.

REFERENCES

E1: Bonvini SJ, Birrell MA, Grace MS, Maher SA, Adcock JJ, Wortley MA, et al. Transient receptor potential cation channel, subfamily V, member 4 and airway sensory afferent activation: Role of adenosine triphosphate. J Allergy Clin Immunol. 2016;138:249–261.e12.

E2: Clarke DL, Belvisi MG, Catley MC, Yacoub MH, Newton R, Giembycz MA. Identification in human airways smooth muscle cells of the prostanoid receptor and signalling pathway through which PGE2 inhibits the release of GM-CSF. Br J Pharmacol. 2004;141:1141–50.

E3: Jia Y, Wang X, Varty L, Rizzo CA, Yang R, Correll CC, et al. Functional TRPV4 channels are expressed in human airway smooth muscle cells. Am J Physiol Lung Cell Mol Physiol [Internet]. 2004; 287: L272-8.

E4: Rousseau E, Cloutier M, Morin C, Proteau S. Capsazepine, a vanilloid antagonist, abolishes tonic responses induced by 20-HETE on guinea pig airway smooth muscle. Am J Physiol Lung Cell Mol Physiol 2005; 288:L460–L470

E5: Baxter M, Eltom S, Dekkak B, Yew-Booth L, Dubuis ED, Maher SA, et al. Role of transient receptor potential and pannexin channels in cigarette smoke-triggered ATP release in the lung. Thorax. 2014;69:1080–9.

E6: Buckley J, Birrell MA, Maher SA, Nials AT, Clarke DL, Belvisi MG. EP4 receptor as a new target for bronchodilator therapy. Thorax. 2011;66:1029–35.

E7: Marrison J, Räty L, Marriott P, O'Toole P. Ptychography--a label free, high-contrast imaging technique for live cells using quantitative phase information. Sci Rep. 2013;3:2369.

E8: Dubuis ED, Belvisi MG, Poushpas S, Flajolet P, Langley K, Birrell MA. A Novel Method For Measuring Contraction Of Primary Human Airway Smooth Muscle Cells. In: Am J Respir Crit Care Med. 2015. p. A1224.

E9: Sanmugalingam D, Wardlaw A, Bradding P. Adhesion of human lung mast cells to bronchial epithelium: evidence for a novel carbohydrate mediated mechanism. 2000. Journal of Leukocyte Biology. 68(1):38-46.

E10: Thorneloe KS, Sulpizio AC, Lin Z, Figuero DJ, Clouse AK, McCafferty GP, Chendrimada TP, Lashinger ES, Girdon E, Evans L, Misajet BA, Demarini DJ, Nation JH, Casillas LN, Marquis RW, Votta BJ, Sheardown SA, Xu X, Brooks DP, Laping NJ, Westfall TD. N-((1S)-1-{[4-((2S)-2-{[(2,4-dichlorophenyl)sulfonyl]amino}-3-hydroxypropanoyl)-1-piperazinyl]carbonyl}-3-methylbutyl)-1-benzothiophene-2-carboxamide (**GSK1016790A**), a novel and potent transient receptor potential vanilloid 4 channel agonist induces urinary bladder contraction and hyperactivity: Part I. 2011. J. Pharmacol Exp Ther. 338(1):410

E11: Cheung M, Bao W, Behm DJ, Brooks CA, Bury MA, Dowdell SE, Eiden HS, Fox RM, Goodman KB, Holt DA, Lee D, Roethke TJ, Willette RN, Xu X, Ye G, Thorneloe KS. Discovery of GSK2193874: An Orally Active, Potent and Selective Blocked of Transient Receptor Potential 4. ACS Med Chem Lett. 2017. 549-554.
