# Supplementary figures and images for: Novel airway smooth muscle–mast cell interactions and a role for the TRPV4-ATP axis in non-atopic asthma
Source: Eur Respir J. 2020 Jul 2;56(1):1901458. doi: 10.1183/13993003.01458-2019 (PMC7330131; doi:10.1183/13993003.01458-2019)

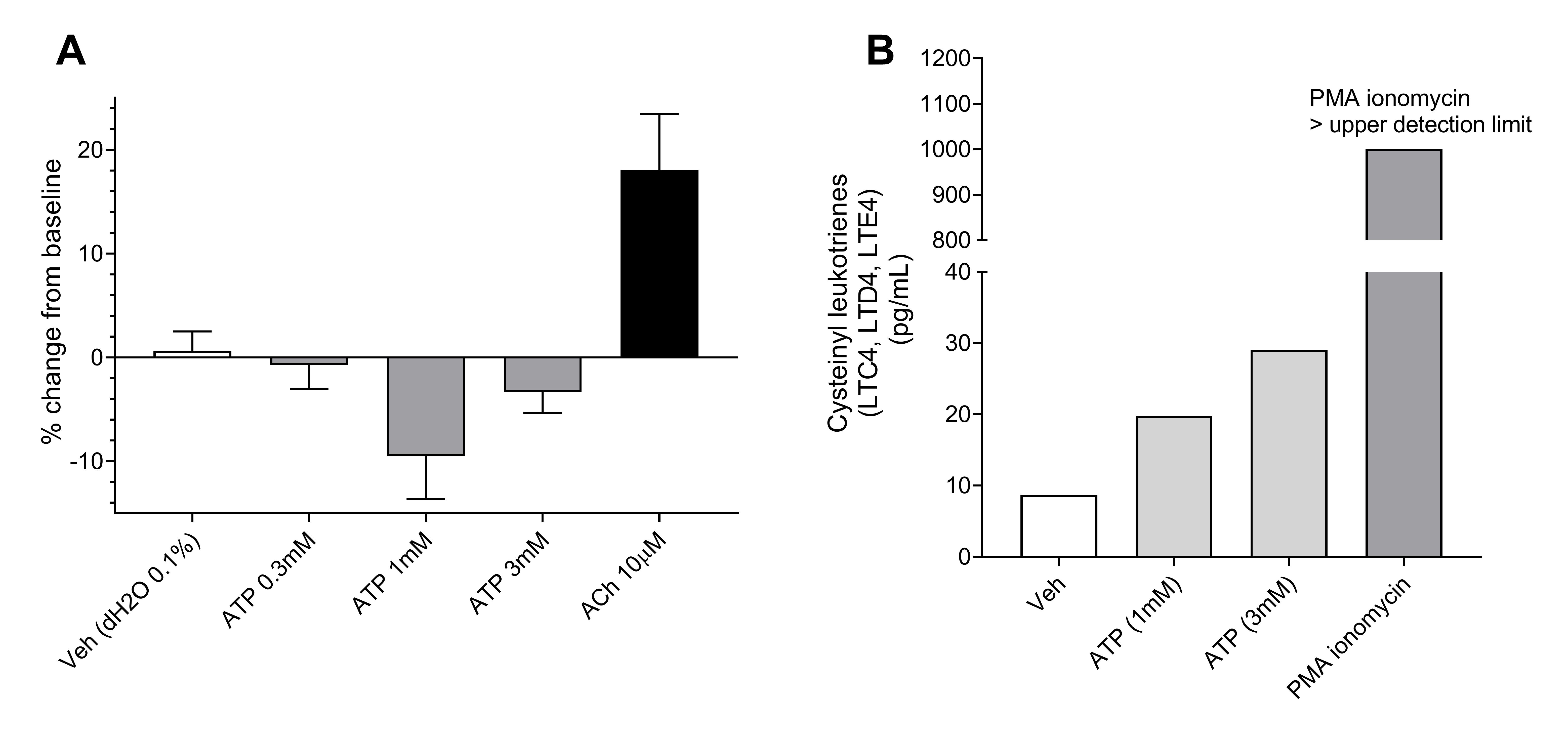

Supplement: Supplementary file 2 [file ERJ-01458-2019.FIGURE_E1.jpg]

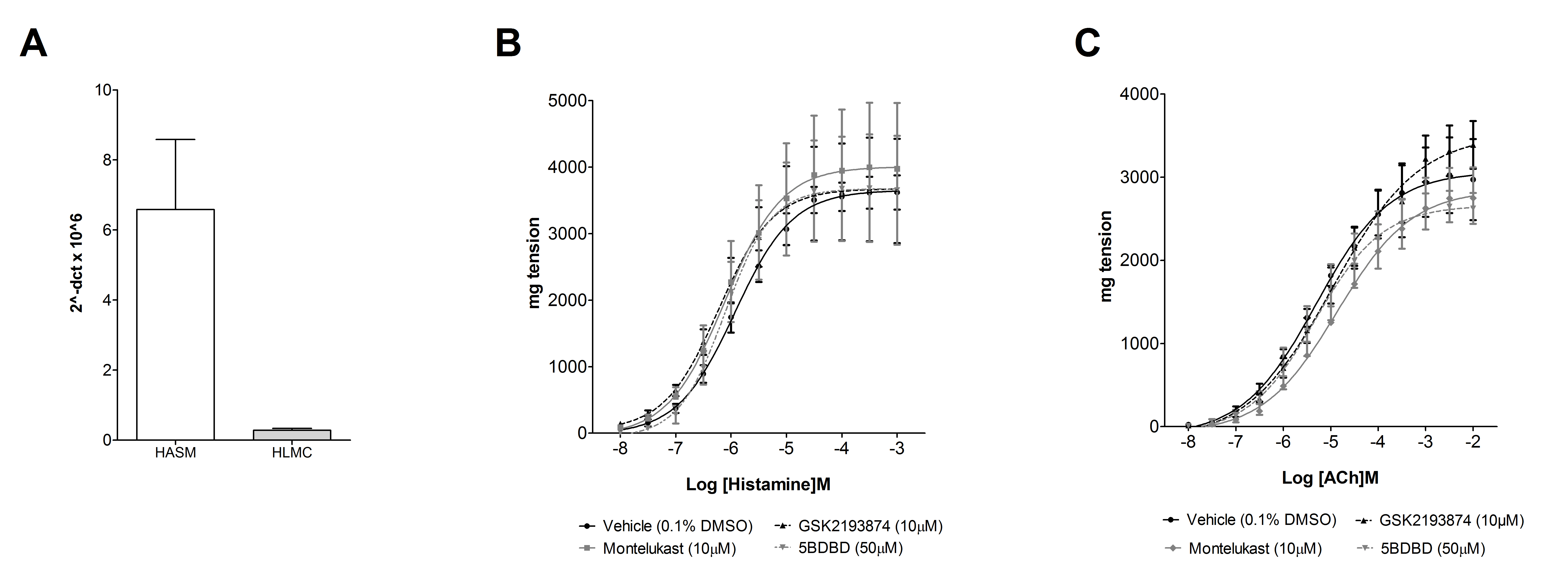

Supplement: Supplementary file 3 [file ERJ-01458-2019.FIGURE_E2.jpg]
