# Supplementary material for: Novel airway smooth muscle–mast cell interactions and a role for the TRPV4-ATP axis in non-atopic asthma
Source: Eur Respir J. 2020 Jul 2;56(1):1901458. doi: 10.1183/13993003.01458-2019 (PMC7330131; doi:10.1183/13993003.01458-2019)
Supplement: Supplementary file 5 [file ERJ-01458-2019.Shareable.pdf]

ORIGINAL ARTICLE
ASTHMA AND BASIC SCIENCE

SHAREABLE PDF

# Novel airway smooth muscle–mast cell interactions and a role for the TRPV4-ATP axis in non-atopic asthma

Sara J. Bonvini[1,2,4], Mark A. Birrell[1,2,4], Eric Dubuis[1], John J. Adcock[1,2], Michael A. Wortley[1], Pauline Flajolet[1], Peter Bradding[3] and Maria G. Belvisi[1,2]

**Affiliations**: [1]Respiratory Pharmacology Group, Airway Disease, National Heart and Lung Institute, Imperial College London, London, UK. [2]Research and Early Development, Respiratory and Immunology, BioPharmaceuticals R&D, AstraZeneca, Gothenburg, Sweden. [3]Dept of Infection, Immunity and Inflammation, University of Leicester University, Institute for Lung Health, Glenfield Hospital, Leicester, UK. [4]Contributed equally.

**Correspondence**: Maria G. Belvisi, Imperial College London, Respiratory Pharmacology, NHLI Sir Alexander Fleming Building, Exhibition Road, London SW7 2AZ, UK. E-mail: m.belvisi@imperial.ac.uk

@ERSpublications
**A technique not previously applied to respiratory research now uncovers important IgE-independent mechanisms involved in human mast cell–airway smooth muscle interactions that may be responsible for the bronchospasm associated with non-atopic asthma** http://bit.ly/2U1n5nT

**Cite this article as:** Bonvini SJ, Birrell MA, Dubuis E, *et al*. Novel airway smooth muscle–mast cell interactions and a role for the TRPV4-ATP axis in non-atopic asthma. *Eur Respir J* 2020; 56: 1901458 [https://doi.org/10.1183/13993003.01458-2019].

This single-page version can be shared freely online.

ABSTRACT Mast cell–airway smooth muscle (ASM) interactions play a major role in the immunoglobulin (Ig)E- dependent bronchoconstriction seen in asthma but less is known about IgE-independent mechanisms of mast cell activation. Transient receptor potential cation channel, subfamily V, member 4 (TRPV4) activation causes contraction of human ASM *via* the release of cysteinyl leukotrienes (cysLTs) but the mechanism is unknown. The objective of the present study was to investigate a role for IgE-independent, mast cell–ASM interaction in TRPV4-induced bronchospasm.

Bronchoconstriction was measured in anaesthetised guinea pigs and contraction of human and guinea-pig airway tissue assessed using isometric tension measurements. Increases in intracellular [$Ca^{2+}$] were imaged using the $Ca^{2+}$-sensitive dye FURA2, and time-lapse ptychography was utilised as a surrogate for contraction of ASM cells.

The TRPV4 agonist GSK1016790A caused contraction *in vivo* in the guinea pig, and in human and guinea-pig tracheal tissue, which was inhibited by the TRPV4 antagonist GSK2193874. GSK1016790A increased $[Ca^{2+}]_i$ and released ATP in human ASM cells without causing contraction. TRPV4 and ATP evoked contraction in isolated tracheal tissue but co-culture experiments indicated a requirement for human lung mast cells. Expression profiling and pharmacological studies demonstrated that mast cell activation was dependent upon ATP activating the P2X4 receptor. Trypsin was shown to evoke contraction of tracheal tissue *via* activation of PAR-2-TRPV4-ATP-cysLT axis indicating the potential disease relevance of this signalling pathway.

TRPV4 activation increases $[Ca^{2+}]_i$ and releases ATP from ASM cells triggering P2X4-dependent release of cysLTs from mast cells resulting in ASM contraction. This study delineates a novel mast cell–ASM interaction and TRPV4 as a driver of IgE-independent mast cell-dependent bronchospasm.

Copyright ©ERS 2020. This version is distributed under the terms of the Creative Commons Attribution Licence 4.0.
